# Caregiver social networks and discontinued support during COVID-19: a multilevel dyadic analysis

**DOI:** 10.1093/geronb/gbag099

**Published:** 2026-06-08

**Authors:** David P Kennedy, Esther M Friedman, James P Murphy, Allison Kirkegaard, Sarah Edgington, Daniela Golinelli, Regina Shih

**Affiliations:** Independent Researcher, Los Angeles, California, United States; RAND Corporation, Santa Monica, California, United States; Institute for Social Research, University of Michigan, Ann Arbor, Michigan, United States; RAND Corporation, Santa Monica, California, United States; RAND Corporation, Santa Monica, California, United States; Institute for Social Research, University of Michigan, Ann Arbor, Michigan, United States; RAND Corporation, Santa Monica, California, United States; RAND Corporation, Santa Monica, California, United States; Center for Health Outcomes and Policy Research, University of Pennsylvania School of Nursing, Philadelphia, Pennsylvania, United States; RAND Corporation, Santa Monica, California, United States; Rollins School of Public Health, Emory University, Atlanta, Georgia, United States; (Social Sciences Section)

**Keywords:** Caregiving, Social capital, Social support, Egocentric networks, Multilevel modeling

## Abstract

**Objectives:**

Family caregivers provide essential support to older adults, but their extended caregiving networks can be strained during crises. This study examines which characteristics of caregivers’ personal networks were associated with discontinued support during the early stage of the COVID-19 pandemic, a period of widespread social disruption.

**Methods:**

We analyzed data from the Care Network Connections over Time study, a longitudinal, nationally representative study of U.S. caregivers. Respondents (*N* = 2,116) reported on support before and during COVID-19. Multilevel logistic regression models assessed associations between discontinued support and network/caregiver characteristics while controlling for clustering within networks.

**Results:**

Support loss occurred among 5.84% of previously supportive network members. Discontinued support was significantly more likely when alters were Hispanic or of “other/multiple” race/ethnicity, aged <18 or >64, or were distant relatives or nonrelatives. Highly connected alters and those providing support to care recipients were less likely to have withdrawn support. At the network level, caregivers with low density, more diverse, or low care-recipient-supporting networks were more vulnerable to support discontinuation.

**Discussion:**

Results demonstrate how caregiver network composition and connectivity shape social resilience under stress. Caregiving networks that have higher “bonding” social capital—densely interconnected and including close relatives and others who provide support to both caregivers and care recipients—may be more resilient due to strong support coordination and communication. Study insights can inform the development of support intervention strategies for caregivers. Findings suggest that promoting bonding capital in caregiver networks may help protect against the loss of critical support during crises.

Caregiving for older loved ones by family and friends plays a critical role in the U.S. public health system (Friedman et al., 2015; Talley & Crews, 2007). This unpaid, often under-recognized caregiving poses financial, emotional, and physical challenges (National Academies of Sciences, Engineering, and Medicine, 2016). As a result, caregivers often share caregiving duties with extended networks of people who help caregivers manage the demands of their role (Friedman & Kennedy, 2021; Marcum et al., 2018).

The COVID-19 pandemic placed unprecedented stress on these caregiving networks, exacerbating existing vulnerabilities (Fingerman & Pillemer, 2021; Park, 2021). Policies designed to reduce the spread of the virus, such as social distancing and shelter-in-place orders, disrupted patterns of in-person informal care. These disruptions may have exacerbated social isolation and loneliness, often experienced by older adults and their caregivers (Leggett et al., 2022; Savla et al., 2021). Studies have highlighted the pandemic’s negative effects on caregivers (Beach et al., 2021) and their support systems (Friedman et al., 2023; Kennedy et al., 2024; Kirkegaard et al., 2024).

Understanding the social networks surrounding the caregiver-care recipient dyad is important. These networks function as a complex system with emergent properties that affect both members of the dyad and other members of the network (Koehly & Manalel, 2023). Different forms of social capital arise from configurations of social relationships and the resources embedded within them. These resources can be mobilized during opportunities or crises (Burt, 2004; Coleman, 1988; Hawkins & Abrams, 2007; Szreter & Woolcock, 2004). Tightly connected networks tend to provide cohesive, supportive “bonding” social capital, while more loosely connected networks provide diverse resources and information. Disruptions, such as the loss of key supporters or shared stressors (e.g., the COVID-19 pandemic), can ripple through caregiving networks. These disruptions may reduce social capital and increase collective strain. Studies of the configuration of caregiving support networks, and how they change under stress, are important because they have implications not only for the caregiver but also for the health outcomes of care recipients (Cornwell & Schafer, 2016). Caregiving support networks played a vital role in caregivers’ ability to continue providing care during the pandemic (Beach et al., 2021), and understanding which caregivers, care recipients, and support network configurations are vulnerable or resilient during public health crises can inform strategies to strengthen family caregiving supports.

Most investigations of caregiving during COVID-19 have either examined the pandemic’s impact on caregivers’ health and well-being without investigating their support networks (Fingerman & Pillemer, 2021; Leggett et al., 2022; Park, 2021; Savla et al., 2021) or focused on the networks solely of care recipients (Brown et al., 2022; Copeland & Liu, 2022). A few small, qualitative, non-representative studies examined caregiving network dynamics (Bristol et al., 2021; Fernández-Peña et al., 2025; Irani et al., 2021; Lightfoot et al., 2021; Savla et al., 2021). Only one study of caregivers to older adults, the Care Network Connections over Time (CNCT) project, has investigated the support networks of caregivers during the pandemic with a large, nationally representative sample (Kennedy et al., 2024). The CNCT project captured the extended support network of caregivers, including those who supported both the caregiver directly, and provided support to the caregivers’ care recipients, or both. Kennedy et al. (2024) analyzed CNCT data to test for associations with the number of people in caregiving support networks who stopped or reduced their support in the early stage of COVID-19. The results demonstrated that caregivers who provided care for individuals with specific conditions (hearing or vision loss), Black caregivers, and those in nursing homes, lost the most support. In contrast, younger caregivers, those from higher-income households, and those who had recently started caregiving experienced fewer losses of support. In terms of network characteristics, larger networks and those with a higher proportion of older members were more vulnerable to losing support. Conversely, networks with densely connected and interacting members were more resilient to reduced support.

These findings suggest different types of factors that may have influenced changes in supportive relationships for caregivers during the early stage of COVID-19. Network member characteristics, such as age or income, may have affected support continuation. Also, the relationship between specific network members and caregivers (e.g., close relatives vs. friends) may have impacted support continuation. However, this study tested associations between counts of lost supportive network numbers and caregivers’ individual and network characteristics. Extending these findings to inferences about individuals within the network based on data at the network level violates the ecological fallacy and may lead to erroneous conclusions (Robinson, 2009). Changes to support relationships may have been impacted by group-level factors that are not associated with characteristics of individuals or relationships. For example, larger networks with more existing aggregate support may have influenced reductions in some network members’ support because they perceived sufficient support from other members of the network. On the other hand, highly interconnected and interacting networks may have influenced stronger social cohesion and enforcement of social norms, which may generate a strong social norm against reductions in support (Coleman, 1990; Kawachi et al., 2008; Milardo et al., 2014). Testing these competing hypotheses at both the group and relationship levels can clarify how network characteristics influence changes in support in different ways across different levels of the network (Vacca, 2018).

Only one published study investigated changes in network support for caregivers during COVID-19 at the relationship level (Fernández-Peña et al., 2025). This study found that network member relationship type (caregivers’ partners vs. professional support) and type of support provided by network members (emotional/informational vs. instrumental) were associated with support changes for caregivers during the pandemic relative to before the pandemic started. The authors acknowledge several limitations to this study, including a convenience sample of caregivers recruited by professionals from health/social centers in one region of Spain, resulting in a non-representative sample, and a small sample size (*N* = 78), which inhibited tests of association between change in support at the relationship level and characteristics of caregivers and their overall support networks.

## Study aims and hypotheses

The primary aim of the current study is to identify factors associated with loss of support from specific network relationships during the early stage of COVID-19, before the availability of vaccines. Informed by social capital theory (Burt, 2004; Coleman, 1988; Hawkins & Abrams, 2007; Szreter & Woolcock, 2004) and findings from previous studies (Fernández-Peña et al., 2025; Kennedy et al., 2024), we tested for associations between characteristics of caregivers, their support networks, and change in support during the pandemic with three main hypotheses. First, we hypothesized that characteristics of individual relationships would drive discontinued support **(H1)**. For example, we expected that caregivers would be less likely to lose support from network members who provide them with high “bonding” social capital, in which support is consistently available, including during times of crises (Coleman et al., 2022; Kawachi et al., 2008; Milardo et al., 2014; Widmer, 2006). Types of network members with high bonding capital include those who are highly connected within the network **(H1a)** and members of the caregivers’ immediate family **(H1b)**. Also, we expect that network members who play more than one supportive role (have role “multiplexity”) (Granovetter, 1973; Wellman & Wortley, 1990), such as supporting the care recipient and the caregiver, have higher bonding capital and are less likely to have discontinued support **(H1c)**.

Second, based on findings in Fernández-Peña et al. (2025) and Kennedy et al. (2024), we expected that the COVID context would disrupt caregiver’s supportive relationships **(H2)**. For example, we expect that age would be associated with the likelihood of discontinuing support during COVID because of the increased risks of face-to-face interactions for older people **(H2a)**. We also expect that caregivers of care recipients who moved from facilities to the community would be more likely to have discontinued support due to reduced face-to-face contact with the caregiver out of fear of indirect contagion **(H2b)**.

Third, we expected that higher bonding social capital would continue to have an independent effect on discontinued support at the overall caregiver and network level, controlling for relationship factors **(H3)**. For example, we expected that the race and income of caregivers/care recipients would be associated with discontinued support **(H3a)**. We also expected that discontinued support would be more likely for minority caregivers/care recipients/network members compared to White caregivers/care recipients/network members **(H3b)**. We also expected that caregivers with higher incomes would be less likely to have network members who discontinued support **(H3c)**. At the network level, we expected that networks with higher overall bonding capital, such as highly interconnected networks **(H3d)** and those with many strong ties or family members **(H3e)**, would be significantly associated with lower loss of support from members of the network. We expected that these associations would be robust in models that controlled for other demographic characteristics of caregivers and network members (e.g., gender). We tested these hypotheses with a multilevel, multivariable analysis of the support networks of caregivers of older adults in the United States during the early stage of the COVID-19 pandemic.

## Method

### Participants and survey period

The current study analyzes data from the first wave of the CNCT study, collected from a nationally representative sample of 2,824 caregivers in the U.S. during the months immediately prior to the introduction of effective COVID-19 vaccines (starting in December 2020) (Kennedy et al., 2024). Respondents were eligible if they were currently caring for someone 50 years or older because of a disability or chronic illness. Details about KnowledgePanel recruitment and screening are available in Supplementary Material (Supplementary Methods). The first author’s IRB reviewed and approved human subject interactions, including the procedure for obtaining consent from participants.

### Measures

#### Caregiver/care recipient characteristics

We tested for associations between discontinued support and a series of respondent and care recipient characteristics available in the CNCT data, which include measures provided by KnowledgePanel (respondent income, education, gender, and geographic area) and newly collected measures. See Supplementary Methods for response categories for each variable included in the analysis. We tested associations between discontinued support and care recipient characteristics, including race/ethnicity, age, gender, relationship to the respondent, where the care recipient lived, care recipient condition, and length of time the care recipient received support from the respondent. Unlike Kennedy et al. (2024), we used a measure of race/ethnicity based on the respondent’s report in the CNCT interview rather than the measure of caregiver race/ethnicity provided by KnowledgePanel. This measure was more precise because it included a separate category of “Asian,” which was not collected by KnowledgePanel. Due to the high collinearity of the race/ethnicity of caregivers and care recipients (an overlap of 88% or higher for similar categories; see Table S2), we selected the measure of the care recipient’s race/ethnicity to include in multivariable models. Similar to Kennedy et al. (2024), we included caregivers having disruptions/challenges/additional caregiving burdens due to COVID-19 in multivariable models.

### Network measures

Details of the personal network interview conducted with CNCT respondents are available in Kennedy et al. (2024). Briefly, after respondents (“egos” in personal network terminology) answered questions about themselves and their care recipient, they were prompted to provide a list of people (their “alters”) with six “name generators” (Marin & Hampton, 2007), such as people they felt close to or discussed important things with, and people who also provided support to the care recipient. Finally, for each unique pair of alters, respondents identified which pairs knew each other and if they contacted each other regularly. Responses to these questions provided the raw data to construct measures of the overall personal (or “egocentric”) network as well as measures of individual alter or ego-alter relationships.

### Alter/ego-alter relationship level

#### Dependent variable: discontinued caregiver support

Respondents were asked about the support provided by each of their alters before the onset of COVID-19 and at the time of the survey. Support was defined as including advice, transportation, errands, housework, financial assistance, or support to the care recipient. The dependent variable “discontinued support” was set to 1 for the alter if the respondent selected “Before the pandemic only” and 0 if the respondent selected “Both now and before the pandemic.” Alters that had not provided support prior to the pandemic were excluded from the multilevel analyses.

#### Alter/ego-alter measures

Respondents indicated what their relationship was with each alter. Alters were classified as close relatives (parent/in-law, spouse, sibling/in-law, children), distant relatives (grandparents, aunts/uncles, grandchildren, other relatives), friends, romantic partners, and other non-relatives. Respondents also classified each alter into a gender category (female, male), an age category (<15, 15-17, 18-24, 25-49; 50-64; >64), and a race/ethnicity category (White, Black, Asian, Hispanic, other/multiple). The age, race/ethnicity, and close relationship variables were dummy coded with the reference categories of age 25-49, White race, and close relatives. We also identified alters who provided any type of support to care recipients at the time of the survey based on responses to a question about the types of support provided by the alter. Respondents also noted which alters knew and interacted with each other regularly. Based on these responses, we calculated alter “degree centrality,” which is a count of the connections each alter has with other members of the network.

#### Egocentric network measures

We generated summary measures of the egocentric network to assess *network composition*. For each alter level measure included in the multivariable models, we calculated the proportions of the alters that egos named with different characteristics by counting the alters with a given characteristic and dividing by the overall number of alters named by the ego. We also assessed *network structure* by calculating network size (the number of alters named by the ego) and egocentric network density (the ratio of existing ties among alters who interacted to the maximum number of potential ties). These measures, which ranged between 0 and 1 in their raw form, were rescaled to range from 0 to 10 to improve the interpretability of the results.

### Analytic sample

Data cleaning resulted in the removal of 83 of the 2,824 respondents because of data quality concerns or excessive patterns of missing data. Of these cases, 2,179 respondents, who named a total number of 11,069 alters, had at least one alter who provided support prior to the start of the pandemic. After removal of 43 complete cases with missing ego-level values (resulting in 188 alters dropped from analysis), and 153 alters with missing values (resulting in an additional 20 cases dropped from analysis), the final multivariable model predicting discontinued support was 2,116 respondents, and 10,728 alters (see Table S4 for details about frequency and % of variables/cases that were removed from the analytic sample due to missingness and Table S5 for a statistical comparison of the 63 respondents removed from the analytic sample with the 2,116 included in the analysis).

#### Data analysis

To test which variables were associated with discontinued support from specific alters (controlling for other variables within and across levels of analysis), we built a multilevel logistic regression model with a one-to-many, dyadic, personal network design (Kenny et al., 2006; Perry et al., 2018; Snijders et al., 1995; Vacca, 2018). Personal network data are inherently multilevel, with measures at the alter and ego-alter relationship level nested within measures at the ego and egocentric network level (Vacca, 2018). A multilevel modeling approach is appropriate to account for both levels of data while controlling for clustering within networks.

We estimated the models using Stata 18 with the “meglm” command for multilevel logistic regression models. Models were estimated with mean-variance adaptive Gauss-Hermite quadrature. To select variables for multivariable models, we ran separate bivariate models testing for associations with discontinued support. We included variables in multivariable models if they were significant at the 95% level in bivariate models. For dummy variables without an obvious reference group (i.e., race/ethnicity), we conducted post hoc analyses comparing categories with different reference groups using a Wald test of the equality of the coefficients. For categories showing statistically different dependent variables, we ran additional multivariable models with new reference groups to generate test statistics.

To account for clustering of alter-level observations within egos, we group-mean centered all alter/dyad-level variables. For all alter/dyads nested within ego *i*, we subtracted the ego-level mean (e.g., proportion of *i*’s network who are male) from the corresponding alter/dyad-level variable (e.g., the dummy variable indicating alter is male). By centering all alter/dyad-level variables on their means, we assure that the centered versions are uncorrelated with the ego-level random effect that represents unobserved ego-level factors. This procedure has been referred to as adaptive mean centering (Raudenbush, 2009) or hybrid fixed-random effects (Allison, 2009; Firebaugh et al., 2013). To account for survey nonresponse and the oversampling of dementia caregivers, analyses were conducted using survey weights. See “Knowledge Panel sampling and weights” in Supplementary Methods for more details.

## Results

Among the 11,069 alters named by 2,179 respondents as having provided them with support prior to the onset of COVID-19, 5.5% stopped providing this support 9 months into the pandemic. Tables 1 and 2 present descriptive statistics and results of bivariate tests for each independent variable that was significantly associated with the dependent variable, discontinued caregiver support. Table 1 presents each ego level association, and network associations are presented in Table 2 (alter/ego-alter measures, network composition and structure).

**Table 1:** Descriptive statistics and odds ratios (95% confidence intervals) of statistically significant bivariate associations^a^ between ego-level measures and stopping providing support to caregivers for all alters who provided support prior to COVID-19 (*N *= 2,179 respondents, 11,069 alters).

Ego-level variables	%	OR	95% CI
**Care recipient race** ^b^			
White (reference)	62.3		
Black	13.8	3.56**	1.86, 6.82
Hispanic	14.0	0.78	0.29, 2.06
Asian	4.5	3.01*	1.06, 8.54
Other/multiple	5.1	1.05	0.33, 3.39
**COVID-19 experiences**			
Care recipient change in residence^b^			
Facility to community	1.8	13.57**	4.54, 40.53
Community to facility	1.4	0.87	0.17, 4.53
Other change	5.3	1.52	0.55, 4.20
No change (reference)	91.4		
**Care recipient condition**			
Old age, aging	48.1	0.54*	0.33, 0.88
Surgery, wounds	6.1	2.61*	1.15, 5.91

*Note.* CI = confidence interval; COVID-19 = coronavirus disease 2019; OR = odds ratio.

a Nonsignificant bivariate tests of association are provided in Supplementary Material.

b Dummy code included in the bivariate model with the reference group.

* *p* < .05.

** *p* < .01.

**Table 2:** Descriptive statistics and odds ratios (95% CI) of statistically significant bivariate associations^a^ between network measures stopping providing support to caregivers for all alters who provided support prior to COVID-19 (*N *= 2,179 respondents, 11,069 alters).

Network variables	%	Mean (*SD*)	OR	95% CI
**Alter race^b^**				
White (reference)^c^	60.8			
Black	16.4		1.12	0.57, 2.19
Asian	3.7		0.48	0.19, 1.19
Hispanic	14.0		1.88	0.99, 3.56
Other/multiple	5.1		2.11*	1.08, 4.14
**Alter age^b^**				
Under 18 years old	3.3		1.61	0.80, 3.26
18-24	6.9		1.05	0.58, 1.94
25-49 (reference)^c^	36.4			
50-64	32.7		1.25	0.93, 1.68
At least 65 years old	20.7		2.57**	1.90, 3.48
**Alter relationship type^b^**				
Close relative (reference)^c^	48.3			
Distant relative	12.7		4.91**	3.38, 7.15
Friend	23.0		3.77**	2.71, 5.26
Romantic partner	1.9		0.28	0.06, 1.26
Other non-relative	11.6		5.42**	3.73, 7.88
**Supports care recipient^b^**				
Does not support	43.6			
Supports	56.4		0.07**	0.05, 0.10
**Alter centrality^b^**				
Degree (strong ties)		3.81 (3.37)	0.62**	0.58, 0.67
**Egocentric network composition (proportion)**				
Race				
White		0.62 (0.43)	0.96	0.90, 1.01
Black		0.13 (0.31)	1.13**	1.05, 1.21
Asian		0.04 (0.16)	1.03	0.88, 1.20
Hispanic		0.13 (0.31)	0.95	0.84, 1.07
Relationship type				
Close relative		0.53 (0.33)	0.78**	0.72, 0.85
Distant relative		0.11 (0.18)	1.20**	1.07, 1.34
Friend or romantic partner		0.25 (0.29)	1.03	0.94, 1.14
Other non-relative		0.08 (0.17)	1.32**	1.17, 1.48
Care recipient support		0.61 (0.34)	0.80**	0.74, 0.87
**Egocentric network structure**				
Density (strong ties)		0.60 (0.29)	0.72**	0.65, 0.80
Network size		5.74 (3.96)	1.15**	1.10, 1.20

*Note*. CI = confidence interval; COVID-19 = coronavirus disease 2019; OR = odds ratio.

a Nonsignificant bivariate tests of association are provided in Supplementary Material.

b Alter-level measures centered on egocentric measures in the bivariate models.

c Dummy code included in the bivariate model with the reference group.

* *p* < .05.

** *p* < .01.

### Descriptive statistics and bivariate tests

Table 1 shows descriptive statistics for ego measures significantly associated with naming an alter that stopped providing support to the ego in bivariate tests (OR, 95% CI). Significant ego-level measures included the care recipient’s race/ethnicity, the care recipient needing to change residence during COVID, and a care recipient who was receiving care because of old age or surgery/wounds. The 2,179 respondents were mostly White (62.3%), with 13.8% Black, 14.0% Hispanic, and around 5% Asian or some other race/multiple races. During COVID, most of the care recipients remained in the same residence (91.4%), while 1.8% moved to the community from living in a nursing facility, 1.4% moved into a facility, and 5.3% experienced another move. Almost half of the respondents were providing care due to the old age of care recipients, and 6.1% were receiving care because of surgery/wounds.

Table 2 provides descriptive statistics (%, mean/*SD*) for each network measure (alter and egocentric) that was significant in bivariate tests of association (OR, 95% CI) with the dependent variable (discontinuing support for the caregiver). Significant associations at the alter-level included alter race/ethnicity, alter age, ego-alter relationship, care recipient support, and network centrality. On average, alters were 60.8% White, 16.4% Black, 14.0% Hispanic, 3.7% Asian, and 5.1% some other race or combination of races. Most alters were in the age range 25-49 (36.4%), 50-64 (32.7%), or 65 or older (20.7%), and most were either close relatives (48.3%), friends (23.0%), or distant relatives (12.7%). Roughly half of alters (56.4%) provided recent support to the care recipient. Alters had an average degree centrality of 3.81.

Egocentric network variables that were significant in bivariate tests included race proportion of alters, the proportion of relationship types, proportion of alters who provided care recipient support, and network structure. The egocentric networks were primarily composed of White alters (mean = 0.62), followed by Black (mean = 0.13), Hispanic (mean = 0.13) and Asian (mean = 0.04). These networks were primarily composed of close relatives (mean = 0.53), friends or romantic partners of the respondent (mean = 0.25), and distant relatives (mean = 0.11). The mean proportion was 0.61 for alters who supported care recipients. The networks averaged 5.74 alters and had an average density of 0.60. Table S1 provides descriptive statistics and results of bivariate tests for variables that were not significantly associated with discontinued support.

### Multivariable, multilevel logistic regression

Table 3 presents the results of the full model testing for associations with odds of support discontinuation. Of the 10,728 alters included in the analytic sample, 626 (5.84%) discontinued support. At the ego-level, the race of care recipients was significantly associated with discontinued support, controlling for alter and network-level factors. Relative to caregivers of White care recipients, alters of Black care recipients (OR = 6.87, 95% CI: 1.49, 31.73) and care recipients categorized as “other/multiple” (OR = 4.20, 95% CI: 1.38, 12.80) had higher odds of naming alters who stopped supporting them during the pandemic. Post hoc analyses showed that caregivers of Hispanic care recipients had lower odds of losing support relative to caregivers of Black care recipients (OR = 0.18; 95% CI: 0.03, 0.97) (see Table S3). Care recipients moving from a nursing facility to the community during COVID were associated with higher odds of discontinued support from any alter (OR = 9.72, 95% CI: 2.83, 33.44). Neither of the care recipient conditions (old age, surgery) included in the model was significantly associated with discontinued support.

**Table 3:** Odds ratios (95% CIs) from multilevel multivariable logistic regression models predicting discontinuation of support (*N *= 2,116 respondents, 10,728 alters, 626 discontinued support).

Variable	Stopped supporting caregiver	
	OR	95% CI
**Caregiver/care recipient characteristics**		
Care recipient race (ref: White)		
Black	6.87*	1.49, 31.73
Hispanic	1.32	0.57, 3.10
Asian	2.31	0.64, 8.25
Other/multiple	4.20*	1.38, 12.80
Care recipient condition		
Old age, aging	0.67	0.42, 1.08
Surgery, wounds	1.17	0.46, 2.97
COVID experiences		
Care recipient moved from facility to community	9.72**	2.83, 33.44
**Alter/ego-alter characteristics** ^a^		
Alter race (ref: White)		
Black	1.16	0.58, 2.35
Asian	0.65	0.25, 1.70
Hispanic	2.63**	1.27, 5.43
Other/multiple/don’t know	4.32**	1.99, 9.36
Alter age (ref: 25-49)		
Under 18 years old	2.37*	1.00, 5.58
18-24	0.97	0.47, 1.98
50-64	1.31	0.93, 1.83
At least 65 years old	2.60**	1.83, 3.70
Supports care recipient	0.10**	0.07, 0.15
Alter–caregiver relationship (ref: Close relative)^b^		
Distant relative	3.11**	2.03, 4.76
Friend	1.32	0.89, 1.95
Romantic partner	0.24	0.05, 1.23
Other non-relative	2.47**	1.61, 3.78
Alter centrality (degree of strong ties)^a^	0.75**	0.69, 0.82
**Network composition (ego)** ^c^		
Proportion race = Black	0.91	0.77, 1.08
Proportion close relatives	0.99	0.88, 1.10
Proportion distant relatives	1.32**	1.13, 1.53
Proportion other non-relatives	1.11	0.97, 1.28
Proportion provide support to care recipient	0.79**	0.72, 0.87
**Network structure (ego)**		
Network size	1.02	0.96, 1.08
Egocentric density (strong ties)^c^	0.79**	0.69, 0.91

*Note*. CI = confidence interval; OR = odds ratio; ref = reference.

a Alter measures centered on egocentric proportion measures.

b Controls for missing alter–caregiver relationship.

c Egocentric measures converted to 0–10 scale in regression models to aid interpretation.

* *p* < .05.

** *p* < .01.

Several alter-level characteristics were significantly associated with discontinued support, controlling for ego and network characteristics. Relative to White alters and controlling for other measures within and across levels, Hispanic alters (OR = 2.63, 95% CI: 1.27, 5.43) and alters in the “other” category (OR = 4.32, 95% CI: 1.99, 9.36) had significantly higher odds of discontinued support. Additional post hoc analyses (see Table S3) revealed that Black (OR=.27, 95% CI: 0.11, 0.70) and Asian (OR=.15, 95% CI: 0.05, 0.48) alters were significantly less likely to discontinue support compared to alters classified as “other/multiple.” Hispanic alters (OR = 3.98, 95% CI: 1.27, 12.91) were significantly more likely to discontinue support compared to Asian alters. Alters under 18 (OR = 2.37, 95% CI: 1.00, 5.58) and 65+ years of age (OR = 2.60, 95% CI: 1.83, 3.70) had higher odds of discontinued support. The odds of discontinued support were significantly lower for alters who supported the care recipient relative to those who did not (OR=.10, 95% CI: 0.07, 0.15). Compared to close relatives, distant relatives (OR = 3.11, 95% CI: 2.03, 4.76) and alters in the “other non-relative” category (OR = 2.47, 95% CI: 1.61, 3.78) had significantly higher odds of discontinued support. Also, higher centrality of the alter was negatively related to discontinued support (OR=.75, 95% CI: 0.69, 0.82).

At the level of the network, egocentric networks composed of higher proportions of alters who were distant relatives (OR = 1.32, 95% CI: 1.13, 1.53) had higher odds of having an alter who stopped providing them support. Egocentric networks with higher proportions of alters who also provided support to care recipients had lower odds of discontinued support (OR = 0.79, 95% CI: 0.72, 0.87). Controlling for network size, egocentric networks that were more interconnected with interacting network members had lower odds of discontinued support (OR = 0.79, 95% CI: 0.69, 0.91). Network size itself was not significantly related to odds of discontinued support.

## Discussion and implications

The current study extends previous research into caregiving networks and how they adjusted their support to caregivers during COVID-19. Investigating extended caregiving networks sheds light on how they respond to stressful circumstances. These dynamics have implications for the health and well-being of caregivers. In addition, changes to caregiver support can have implications for those they care for as well. Research that informs policies relevant to caregiving in the United States is essential because of the reliance on family and friends throughout the health system (Talley & Crews, 2007). The extended network of people connected to caregivers is a social system that provides essential day-to-day care and is also available to provide support in times of extreme change or crisis. Caregivers of family and friends are vulnerable to experiencing social isolation and loneliness during an extreme crisis, such as a pandemic (Hajek et al., 2021), causing stress beyond their ability to adjust. Identifying which caregivers, care recipients, and caregiving systems are most vulnerable to social disruption is important. These insights can inform policies affecting the health of both care recipients and caregivers (Cornwell & Schafer, 2016).

These findings build on previous studies of change in caregiver support during COVID-19. The current analysis extends Kennedy et al. (2024) by testing associations with changes to support relationships that were suggested by associations with changes in aggregate network support. The current study uses multilevel modeling to test for precise relationship outcomes controlling for factors at other levels, similar to Fernández-Peña et al. (2025) but with a larger, nationally representative sample. Controlling for network and alter-level factors, several findings are consistent with previous findings. For example, caregivers of care recipients who moved from a facility to the community (including living with the caregiver) during COVID-19 were significantly more likely to experience discontinued support (H2b). This finding supports the hypothesis that this move affected caregiver support at the individual relationship level (H1). It is also consistent with findings reported in Fernández-Peña et al. (2025), which found that caregivers reported losing support previously provided in face-to-face interactions with alters during periods of COVID-19 lockdown out of fear of contagion. A move from a facility to the community may have led to caregivers and alters reducing in-person contact to avoid indirectly increasing the risk of infection to the care recipient. Although this strategy may have helped reduce the spread of infection, it also may have introduced a barrier for caregivers to receive the same in-person support they received prior to the pandemic. The current study also found a significant (negative) association between egocentric network density and loss of support for caregivers (H3d). This finding supports the hypothesis that dense networks are high in bonding capital and, therefore, may provide resilience against network members discontinuing support during a crisis (Kawachi et al., 2008). Although the Fernández-Peña et al. (2025) study did not find a significant association between network structure and change in support—likely because of a small sample size—the current study’s large, nationally representative sample provided sufficient multilevel data to fully test this association while controlling for measures across data levels.

The most novel findings in the current study are from explicit tests of association between discontinued support and alter/relationship variables (H1). Support for care recipients was associated with continued support for caregivers at both the alter and the network level. In other words, those who were providing support for care recipients at the time of the survey were unlikely to have discontinued support for caregivers. Also, alters in networks with high proportions of care recipient supporting alters were more likely to continue caregiver support, controlling for alter level care recipient support (H3e). This suggests that shared caregiving roles may signal strong ties between alters and caregivers. In addition, high levels of care recipient support across the network may indicate resilient support systems during a crisis (Granovetter, 1973; Wellman & Wortley, 1990). The findings also show that connectivity among members of the support network is a possible indicator of support resilient to a crisis. Controlling for overall density, alter level connectivity was associated with sustained support for caregivers (H1a). These indications of high bonding capital may buffer against lost support for caregivers during a crisis (Coleman et al., 2022; Kawachi et al., 2008; Milardo et al., 2014; Widmer, 2006). Networks in which alters have many strong ties may promote support coordination, communication, and resilience during crises.

In contrast to Fernández-Peña et al. (2025), which found no significant associations between caregiver demographics and support during COVID-19, several findings related to race/ethnicity were significant (H3a). These findings concur with Kennedy et al. (2024), but offer unique insights due to more precision in the measurement of race/ethnicity. Black and Hispanic races were associated with lost support, but at different levels of analysis. Alters with Hispanic race/ethnicity had a higher probability of no longer providing support relative to White alters, whereas caregivers of care recipients with Black race/ethnicity had significantly higher odds of discontinued support from any alter, relative to caregivers of White care recipients. Caregivers of Hispanic care recipients, relative to Black care recipients, were less likely to lose support. It is unclear why these dynamics would operate differently for Black and Hispanic care recipients and/or their caregivers. Also, the confidence interval for several of the significant associations between caregiver/care recipient race/ethnicity and lost alter support were very wide, particularly those with Black race/ethnicity (OR 95% CI: 1.49-31.73). Therefore, we recommend caution when interpreting these results. We also recommend additional research that tests associations between ethnic differences and support continuity with larger subgroup samples. The “other” race/ethnicity category was associated with stopped caregiver support at both the network and alter levels. These findings may be another example of the association between low bonding capital and support resiliency (Kawachi et al., 2008) because resources are more likely to be shared within social groups whose members are alike (“homophily”) in terms of their social identity, such as class or race (H1a, H3d). The “other” classification, which groups together a mixture of different low prevalence groups, is an indication of diversity.

The current study also provides more insight into family dynamics related to change in support. In contrast to Kennedy et al. (2024), the current study disaggregated close and distant relatives at both the alter and network level and found that distant relatives and non-relatives were more likely than close relatives to stop supporting caregivers (H1e). There was a similar pattern of associations at the overall network composition level. These findings emphasize the importance of distinguishing between close, distant, and non-relatives in research into support consistency during crisis. Extended family members are less frequently caregivers compared to close relatives (Nichols et al., 2011; Wolff et al., 2016), are understudied, and are sometimes assumed to be sources of latent caregiving support during crises (Roberto & Savla, 2022). However, the current findings demonstrate that close relatives are more reliable for continuing support to caregivers during crises than distant relatives or non-relatives.

The current study also provides new insight into how the age of members of the support network are associated with loss or continuation of support during the pandemic. At the alter level, the youngest (18 years old and younger) and oldest (65+) alters were more likely to stop supporting caregivers. It is likely that older alters reduced their support due to greater vulnerability to COVID infections (H2a). Alters in their teenage years were vulnerable to stressful disruptions in their social lives and education, and to experiencing mental health problems during the pandemic (Zolopa et al., 2022). Therefore, they may have been unable to provide any support to parents who were caring for older adults. Having higher percentages of young alters who were previously supportive was also associated with a higher probability of any alter stopping providing support to caregivers. Support that was previously available for caregivers may have shifted instead to vulnerable teens in the network, which provided resilience against increased stress during COVID (Jost et al., 2023). These findings provide some support for concerns about unique stressors experienced by caregivers in the “sandwich” generation (Lei et al., 2023) and suggest that support for caregivers from members of the network may shift away from them toward other vulnerable network members during times of crises, which may compound the stress of caregiving during these times.

Several significant associations with overall lost support were reported in Kennedy et al. (2024), such as caregiver income, length of time caregivers have been providing care, the co-residence of caregivers and their care recipients, residence of care recipients in care facilities, and caregivers’ experience of travel restrictions during COVID-19, were not significantly associated with discontinued support measured at the relationship level (H3). It is possible that these associations are only network-level findings that are not significant when controlling for alter-level factors. This underscores the problem of not accounting for relationship-level associations when testing for associations among group-level variables only.

This study has many strengths and some notable limitations. The current analyses come from the CNCT study, which is the largest study of the personal networks of caregivers providing support to older adults in the United States. The personal network design enables a multilevel analysis approach that allows for hypothesis testing at multiple levels of the network. CNCT is the only nationally representative study to use this analysis approach to understand support for caregivers during a health crisis. We assessed changes in the support relationships among individual members of the extended caregiver support networks without collapsing relationship data into overall network summary variables. Despite these strengths, there are some limitations that are worth noting. The dependent variable is based on retrospective accounts of changes in support. Perceptions of relationships in the past may be biased toward current experiences. Although the analyses address change in support over time, the data are from cross-sectional data collection and are not as reliable as repeated measures at different time points. In addition, the measure of caregiver support before the pandemic does not distinguish among different domains of support, which limited our ability to further explore important contexts relevant to discontinued support. Consequently, we are unable to distinguish between alters who discontinued providing minor and/or occasional support before COVID-19 from those who discontinued providing regular and significant support. Also, the reports about network characteristics and the care recipient are from only one member of the support network. Reports from multiple caregivers would provide an enhanced understanding of the dynamics of the caregiving network. The selection of independent measures for the multivariable model was based on statistical significance in bivariate tests rather than a theoretically informed selection approach. This may have introduced unknown bias due to the exclusion of theoretically important measures. Finally, the complete case analysis approach resulted in the exclusion of cases based on missing data. Although the number of cases removed was small (<3%), this analytic approach may have introduced unknown bias.

This study provides valuable insights into how caregiving networks responded to a health emergency. Findings highlight the importance of network density, multiplexity, and social capital for sustaining caregiving support during times of crisis. Minority caregivers and those with fewer resources were more likely to lose support, underscoring the need for policies that address systemic inequalities in caregiving networks. Close family ties and interconnected networks were critical for maintaining support, while age-related vulnerabilities influenced the availability of support. Future research should include longitudinal data and multiple informants within caregiving networks. These insights can help inform strategies to strengthen social support to family caregivers to increase their social resilience in the face of future public health emergencies.

## Supplementary Material

gbag099_Supplementary_Data

## Data Availability

The data underlying this article will be shared in a repository that is currently in progress. Contact the first author, Dr. David Kennedy, or the PI of the project grants, Dr. Esther M. Friedman, with any queries.
