# Supplementary material for: Caregiver social networks and discontinued support during COVID-19: a multilevel dyadic analysis
Source: J Gerontol B Psychol Sci Soc Sci. 2026 Jun 8;81(8):gbag099. doi: 10.1093/geronb/gbag099 (PMC13353213; doi:10.1093/geronb/gbag099)
Supplement: Supplementary file 1 [file gbag099_supplementary_data.docx]

***The Journals of Gerontology, Series B: Psychological Sciences and Social Sciences* Supplementary Material: Kennedy et al. Caregiver social networks and discontinued support during covid-19: a multilevel dyadic analysis.**

**Supplementary Methods**

**1. Knowledge Panel sampling and weights**

Participants were recruited from KnowledgePanel, a probability‐based, nationally representative web panel maintained by The Ipsos Public Affairs. Eligibility of KnowledgePanel members for sampling included (1) at least 18 years old, (2) resided in the United States, and (3) did not reside in an institution. We recruited a random sample of panelists and also an oversample of panelists who had indicated in prior KnowledgePanel surveys that they provided care to someone with dementia.

Initial invitations were sent to 28,592 individuals on December 17, 2020 with four subsequent email reminders sent over the next 2 weeks. Of those invited, 17,477 responded to the following screening question:

“In the past 12 months, were you helping out or looking after a family member or friend 50 years or older due to a chronic illness or disability? This may include help with tasks such as bathing, feeding, giving medicines, transportation, household chores, handling finances, or arranging for services. You do not necessarily need to live with them.”

If the panelist responded “Yes,” they were then asked if they were “currently” providing this support. Around 22% of the individuals who completed the screening questions (3900) had provided care to an older adult within the past 12 months and 16% (2824) were currently providing that care. All surveys were completed by January 4, 2021.

Survey weights were developed to adjust for survey nonresponse as well as the dementia oversample. Ipsos constructed study-specific weights so that respondents would be representative of the national caregiver population. Weight construction was a two-stage process. In the first stage, “screening weights” benchmarked to sociodemographic measures from the 2019 American Community Survey were used to construct the distribution of characteristics for the caregiver population. The demographic attributes included age, gender, race-ethnicity, education, household income, and Census region. Then, based on this distribution, sample-specific weights were constructed to adjust for the dementia caregiver oversample.

**2. Response categories for all variables included in analysis**

1. Caregiver Household Income
   a. Less than $40,000
   b. $40,000 to $100,000
   c. Over $100,000
2. Caregiver Education
   a. Less than high school
   b. High school
   c. Some college
   d. Bachelor’s degree or higher
3. Caregiver Gender
   a. Female
   b. Male
4. Caregiver Geographic Area
   a. Urban
   b. Rural
5. Caregiver State of Residence
   a. (Specific State of residence)
6. Caregiver Age
   a. (numeric response)
7. Caregiver Marital Status
   a. Married
   b. Widowed
   c. Divorced
   d. Separated
   e. Never married
   f. Living with partner
8. Caregiver Employment Status
   a. Working - as a paid employee
   b. Working - self-employed
   c. Not working - on temporary layoff from a job
   d. Not working - looking for work
9. Care recipient race
   a. White
   b. Black or African American
   c. American Indian or Alaska Native
   d. Asian
   e. Pacific Islander
   f. Hispanic
   g. Some other race/ethnicity
   h. Multiple races/ethnicities
10. Negative caregiver COVID experiences
    a. Stopped visiting friends or family
    b. Stopped visiting older relatives
    c. Began working remotely
    d. Canceled travel plans
    e. Became sick with coronavirus
    f. Lost a job or were furloughed
    g. Children who had been living at college moved back home
    h. Children in kindergarten through 12th grade stopped going to school in person
11. Care recipient condition requiring caregiving
    a. Alzheimer’s, confusion, dementia, forgetfulness
    b. Arthritis
    c. Back problems
    d. Blood pressure, hypertension
    e. Brain damage or injury
    f. Broken bones
    g. Cancer
    h. Coronavirus (COVID-19)
    i. Developmental or intellectual disorder/disability
    j. Diabetes
    k. Feeble, unsteady, falling, frail
    l. Hearing loss, deafness
    m. Heart disease, heart attack
    n. Lung disease, emphysema, COPD
    o. Mental illness, emotional illness, depression
    p. Mobility problem, can’t get around
    q. Old age, aging
    r. Parkinson’s
    s. Stroke
    t. Substance, drug, or alcohol abuse
    u. Surgery, wounds
    v. Vision loss, blindness, can’t see well
    w. Other
12. Care recipient age
    a. (numeric response)
13. Care recipient gender
    a. Male
    b. Female
    c. Other
14. Care recipient change in residence during COVID
    a. Their residence has not changed
    b. They used to live in a group home or institution but moved in with you
    c. They used to live in a group home or institution but moved in with someone else
    d. They used to live in a group home or institution but moved into their own home in the community
    e. They moved into a group home or institution
    f. They moved, but none of the situations above apply
15. Length of care
    a. Less than 6 months
    b. Between 6 months and 1 year
    c. 1-3 years
    d. 4-6 years
    e. 7-9 years
    f. 10 or more years
16. Travel distance between caregiver and care recipient
    a. Less than 20 minutes
    b. Between 20 minutes and an hour
    c. 1-2 hours
    d. More than 2 hours
17. Alter race
    a. White
    b. Black or African American
    c. American Indian or Alaska Native
    d. Asian
    e. Pacific Islander
    f. Hispanic
    g. Some other race/ethnicity
18. Alter age
    a. Under 15 years old
    b. 15-17
    c. 18-24
    d. 25-49
    e. 50-64
    f. At least 65 years old
19. Alter gender
    a. Male
    b. Female
    c. Other
20. Alter’s relationship to caregiver (if “Yes, a relative”)
    a. Grandparent or great-grandparent
    b. Parent or step-parent
    c. Parent-in-law (spouse’s parent)
    d. Aunt or uncle
    e. Spouse or partner
    f. Sibling or step-sibling
    g. Sibling-in-law (sibling’s spouse)
    h. Child or step-child
    i. Grandchild
    j. Other relative
21. Alter’s relationship to caregiver (if “No, not a relative”)
    a. Friend
    b. Boyfriend, girlfriend, or romantic partner
    c. Coworker
    d. Neighbor
    e. Member of your religious community or place of worship
    f. Member of a social support group you belong to
    g. Paid helper
    h. Outside service provider
    i. Other
    j. I don’t know them personally
22. Alter support to caregiver
    a. Both now and before the pandemic
    b. Now only
    c. Before the pandemic only
    d. Has not provided support
23. Type of alter support to care recipient
    a. Advice, encouragement, moral or emotional support
    b. Transportation, errands, or shopping
    c. Housework, yard work, repairs, or other work around the house
    d. Money or financial assistance
    e. Managing finances (such as bills or insurance paperwork)
    f. Personal care (such as help with bathing, dressing, and eating)
    g. Medical/nursing tasks (such as giving medicines or monitoring blood pressure)
    h. Joining doctor’s visits or communicating with health care professionals about their care
    i. Arranging or supervising paid services (such as nurses, aides, Meals on Wheels, or other services)
    j. Advocating for them with government agencies, community services, or health care providers (such as helping them get Medicaid benefits)
24. Relationship between caregiver and each alter
    a. We don’t know each other
    b. Know, but no regular contact
    c. Know, with regular contact
25. Relationship between care recipient and each alter
    a. They don’t know each other
    b. Know, but no regular contact
    c. Know, with regular contact
    d. I’m not sure
26. Relationship between each alter
    a. They don’t know each other
    b. Know, but no regular contact
    c. Know, with regular contact
    d. I’m not sure

**Supplementary Tables**

**Supplementary Table 1. Descriptive statistics and odds ratios of variables not significant in bivariate tests**

|  | **Caregivers (n =2,179)** | |
| --- | --- | --- |
|  | **Alters (n = 11,069)** | |
|  | **% / Mean (SD)** | **OR (95% CI)** |
| **Ego Level Variables** |  |  |
| Caregiver Gender |  |  |
| Male | 41.3 | 0.71 (0.41, 1.23) |
| Female | 58.7 |  |
| Caregiver Age |  |  |
| 18-29 | 16.9 | 1.03 (0.41, 2.59) |
| 30-44 | 23.0 | 0.98 (0.47, 2.02) |
| 45-59 | 30.7 |  |
| 60+ | 29.3 | 0.96 (0.56, 1.66) |
| Caregiver Marital Status |  |  |
| Unmarried | 36.3 | 1.18 (0.69, 2.01) |
| Married or living with partner | 63.7 |  |
| Caregiver Employment Status |  |  |
| Not working | 37.1 | 1.05 (0.62, 1.78) |
| Working | 62.9 |  |
| Caregiver Education |  |  |
| No college degree | 71.6 | 0.70 (0.44, 1.11) |
| College degree or higher | 28.4 |  |
| Caregiver yearly income |  |  |
| Less than $40K (reference) | 37.5 |  |
| $40-100K | 39.8 | 0.63 (0.35, 1.12) |
| >$100K | 22.7 | 0.69 (0.35, 1.32) |
| Covid Experience |  |  |
| Children in K-12 stopped in-person school | 25.0 | 1.11 (0.61, 2.03) |
| Other negative covid experience | 40.7 | 1.06 (0.63, 1.76) |
| Caregiver lives in urban area | 87.6 | 1.21 (0.58, 2.53) |
| State COVID restriction policies |  |  |
| Low | 22.2 |  |
| Moderate | 41.9 | 1.11 (0.57, 2.15) |
| High | 36.0 | 1.07 (0.55, 2.09) |
| Care Recipient Conditions |  |  |
| Arthritis | 32.0 | 1.05 (0.61, 1.81) |
| Mental illness, emotional illness, depression | 13.5 | 0.94 (0.48, 1.84) |
| Other conditions | 26.6 | 1.53 (0.87, 2.68) |
| Care Recipient Age |  |  |
| CR age 50 to 64 | 24.3 | 1.69 (0.84, 3.38) |
| CR age 65 to 79 | 39.0 |  |
| CR age 80+ | 36.6 | 0.94 (0.53, 1.64) |
| Care Recipient Gender |  |  |
| Male | 35.3 | 1.10 (0.65, 1.87) |
| Female | 64.7 |  |
| Length of Care |  |  |
| Less than 1 year | 25.2 |  |
| 1 to 3 years | 35.9 | 0.97 (0.50, 1.90) |
| 4 to 9 years | 24.0 | 0.62 (0.28, 1.34) |
| 10 or more years | 14.9 | 0.84 (0.35, 2.04) |
| Travel distance between Caregiver & Care Recipient |  |  |
| Live together | 39.4 |  |
| Less than 20 minutes | 34.5 | 0.67 (0.36, 1.22) |
| Between 20 minutes and an hour | 18.6 | 1.38 (0.69, 2.76) |
| 1-2 hours | 3.2 | 2.13 (0.82, 5.52) |
| More than 2 hours | 4.3 | 0.47 (0.16, 1.38) |
| **Network Level Variables** |  |  |
| Network Composition (average proportion) |  |  |
| Female | 0.59 (0.26) | 0.99 (0.89, 1.10) |
| **Alter Level Variables** |  |  |
| Female Gender | 60.3 | 1.11 (0.89, 1.39) |

**Supplementary Table 2. Caregiver and care recipient race correspondence**

| **Caregiver and Care Recipient Race Correspondence in the Sample for Model Predicting Support to Caregiver** | | | | |
| --- | --- | --- | --- | --- |
|  | **Caregiver Race/ethnicity** | | | |
|  | **White, Non-Hispanic** | **Black, Non-Hispanic** | **Hispanic** | **Other/multiple, Non-Hispanic** |
| Care recipient race |  |  |  |  |
| White | 7,236 | 80 | 296 | 297 |
|  | 95.16 | 6.16 | 22.32 | 37.79 |
| Black | 52 | 1,173 | 28 | 86 |
|  | 0.68 | 90.30 | 2.11 | 10.94 |
| Hispanic | 128 | 13 | 867 | 1 |
|  | 1.68 | 1.00 | 65.38 | 0.13 |
| All others | 188 | 33 | 135 | 402 |
|  | 2.47 | 2.54 | 10.18 | 51.15 |
|  |  |  |  |  |
| Caregiver & care recipient race overlap: | | 87.86% |  |  |

**Supplementary Table 3. Coefficients from full discontinued support model, modifying race omitted categories**

| **Alter Race** |  |  |  |
| --- | --- | --- | --- |
| **Asian as reference** |  |  |  |
|  | **Coefficient** | **95% CI** | **p-value** |
| Alter race: White (centered) | 1.547 | [0.593, 4.036] | 0.372 |
| Alter race: Black (centered) | 1.802 | [0.573, 5.662] | 0.314 |
| Alter race: Hispanic (centered) | 4.053 | [1.273, 12.909] | 0.018 |
| Alter race: Other/multiple/dk (centered) | 6.633 | [2.069, 21.267] | 0.001 |
|  |  |  |  |
| **Other as reference** |  |  |  |
| Alter race: White (centered) | 0.233 | [0.108, 0.505] | 0.000 |
| Alter race: Black (centered) | 0.272 | [0.106, 0.695] | 0.007 |
| Alter race: Asian (centered) | 0.151 | [0.047, 0.483] | 0.001 |
| Alter race: Hispanic (centered) | 0.611 | [0.231, 1.618] | 0.321 |
|  |  |  |  |
| **Care Recipient Race** |  |  |  |
| **Black as omitted category** |  |  |  |
| White | 0.144 | [0.032, 0.657] | 0.012 |
| Hispanic | 0.181 | [0.034, 0.970] | 0.046 |
| Asian | 0.343 | [0.049, 2.413] | 0.282 |
| Other or multiple | 0.598 | [0.107, 3.347] | 0.559 |
|  |  |  |  |

**Supplementary Table 4. Counts of missing values of different types of variables and complete cases or alters dropped from analysis due to missingness in complete case analysis**

|  | **# missing** | **% missing** |
| --- | --- | --- |
| Alter race/ethnicity | 152 | 1.37 |
| Alter age | 61 | 0.55 |
| Care recipient Health Conditions | 46 | 0.42 |
| Care recipient race/ethnicity | 63 | 0.57 |
| Care recipient moved from facility to community | 84 | 0.76 |
| Alters with one or more missing variables | 341 | 3.08 |
| Total cases with any missing data | 86 | 3.95 |
| Alters excluded due to missing one or more respondent-level variables | 188 | 1.70 |
| Total networks dropped entirely due to missing ego data | 43 | 1.97 |
| Alters missing 1+ alter-level vars (after dropping those missing ego-level) | 153 | 1.38 |
| Alters missing one or more alter-level variables resulting in whole case drop | 116 | 1.05 |
| Alters missing one or more alter-level variables resulting in alter drop | 37 | 0.33 |
| Total networks dropped entirely due to missing alter data | 20 | 0.92 |
| Total networks with some alters dropped due to missing alter data | 23 | 1.06 |

**Supplementary Table 5. Comparison of characteristics of caregivers included in analytic sample with caregivers excluded due to missing values at the respondent or alter level**

|  | **Case included in analysis (N=2,116)** | **Case excluded due to missing data (N=63)** | **Chi-square test *p* < .05** | |
| --- | --- | --- | --- | --- |
| Gender |  |  |  | |
| Male | 42.34 | 32.52 |  | |
| Female | 57.66 | 67.48 |  | |
| Age |  |  |  | |
| 18-29 | 16.60 | 28.95 |  | |
| 30-44 | 23.05 | 28.81 |  | |
| 45-59 | 30.71 | 24.55 |  | |
| 60+ | 29.65 | 17.69 |  | |
| Race/ethnicity |  |  | * | |
| White, Non-Hispanic | 58.91 | 37.09 |  | |
| Black, Non-Hispanic | 13.26 | 16.32 |  | |
| Other, Non-Hispanic | 6.18 | 3.55 |  | |
| Hispanic | 19.40 | 41.45 |  | |
| 2+ Races, Non-Hispanic | 2.25 | 1.60 |  | |
| Marital status |  |  | * | |
| Married/Cohabiting | 64.31 | 59.19 |  | |
| Widowed | 2.61 | 1.43 |  | |
| Divorced | 10.25 | 1.21 |  | |
| Separated | 1.91 | 2.49 |  | |
| Never married | 20.91 | 35.68 |  | |
| Education |  |  |  | |
| Less than high school | 13.03 | 21.37 |  | |
| High school | 27.04 | 25.85 |  | |
| Some college | 31.99 | 32.55 |  | |
| Bachelor's degree or higher | 27.95 | 20.23 |  | |
| Employment status |  |  | * | |
| Employee | 52.13 | 47.89 |  | |
| Self-employed | 11.37 | 1.10 |  | |
| Looking for work/laid off | 5.67 | 16.71 |  | |
| Retired or disabled | 24.90 | 26.33 |  | |
| Other | 5.92 | 7.97 |  | |
| Household income |  |  |  | |
| Less than $40,000 | 37.05 | 39.51 |  | |
| $40,000 to $100,000 | 40.36 | 35.40 |  | |
| Over $100,000 | 22.59 | 25.09 |  | |
| Any kids <18 in household |  |  |  | |
| No child <18 in HH | 71.47 | 71.48 |  | |
| Child <18 in HH | 28.53 | 28.52 |  | |
| Urbanicity of caregiver |  |  |  | |
| Non-Metro | 12.69 | 9.03 |  | |
| Metro | 87.31 | 90.97 |  | |
| Region of residence |  |  |  | |
| New England | 3.98 | 3.90 |  | |
| Mid-Atlantic | 13.05 | 14.62 |  | |
| East-North Central | 13.37 | 15.64 |  | |
| West-North Central | 4.69 | 4.00 |  | |
| South Atlantic | 20.42 | 12.57 |  | |
| East-South Central | 5.56 | 2.57 |  | |
| West-South Central | 16.66 | 23.72 |  | |
| Mountain | 6.11 | 6.98 |  | |
| Pacific | 16.16 | 15.99 |  | |
| * *p* <.05 difference between CGs with and without missing data in network | | | |  |
